# *53bp1* mutation enhances *brca1* and *bard1* embryonic lethality in *C. elegans*

**DOI:** 10.17912/micropub.biology.000934

**Published:** 2023-07-29

**Authors:** Sara Hariri, Qianyan Li, JoAnne Engebrecht

**Affiliations:** 1 Biochemistry, Molecular, Cellular and Developmental Biology Graduate Group, Department of Molecular and Cellular Biology, University of California, Davis

## Abstract

In mice, mutation of *brca1* results in embryonic lethality, which is partially suppressed by *53bp1* mutation. In contrast, mutation of the *C. elegans* BRCA1 ortholog, *brc-1 ,* or its binding partner, *brd-1* , lead to only mild embryonic lethality. We show that in *C. elegans* , *brc-1* and *brd-1* embryonic lethality is enhanced when *53bp1* ortholog, *hsr-9* , is also mutated. This is not a consequence of activating *polq-1* -dependent microhomology-mediated end joining, as *polq-1* mutation does not suppress embryonic lethality of *hsr-9 ; brc-1* mutants. Together, these results suggest that BRC-1 - BRD-1 and HSR-9 function in parallel pathways and do not act antagonistically as in mammals.

**Figure 1. Embryonic lethality in different allele combinations of brc-1, brd-1, hsr-9, and polq-1:**
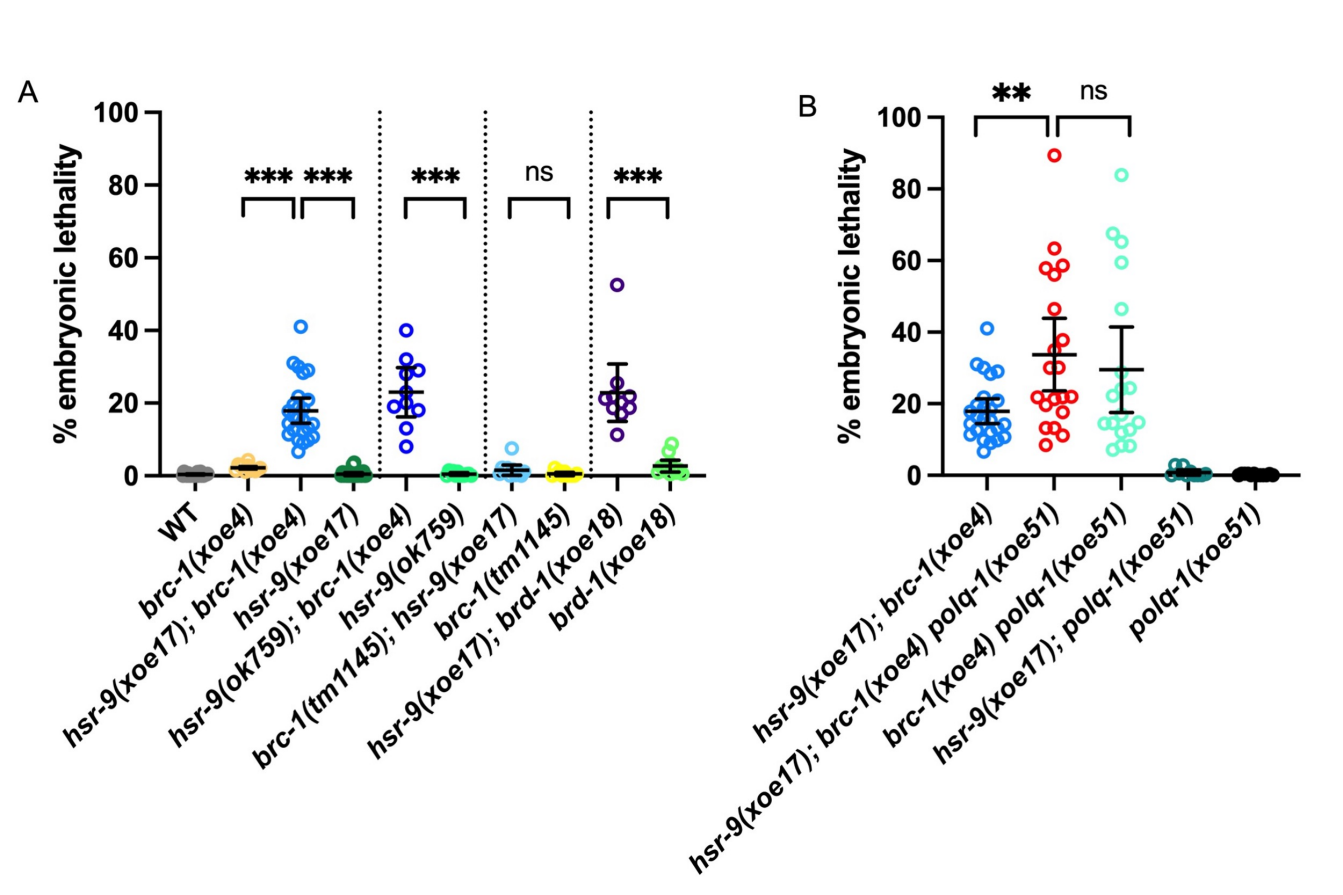
A) Embryonic lethality in wild type (26), *brc-1(xoe4)* (12), *hsr-9(xoe17);* *brc-1(xoe4)* (25), *hsr-9(xoe17)* (23) *, hsr-9(ok759); brc-1(xoe4)* (12) *, hsr-9(ok759)* (12) *, hsr-9(xoe17); brc-1(tm1145)* (11) *, brc-1(tm1145)* (11) *, hsr-9(xoe17); brd-1(xoe18)* (10), and *brd-1(xoe18)* (12) animals. Number of animals examined are in paratheses. B) Embryonic lethality in *hsr-9(xoe17);* *brc-1(xoe4)* (25), *hsr-9(xoe17);* *brc-1(xoe4) polq-1(xoe51)* (20) *, brc-1(xoe4) polq-1(xoe51)* (18) *, hsr-9(xoe17); polq-1(xoe45)* (10) and *polq-1(xoe51)* (12). Mean and 95% Confidence Interval shown; *** p < 0.001; ** p < 0.01; ns = not significant by Mann-Whitney.

## Description

BRCA1-BARD1 is an essential E3 ubiquitin ligase that functions as a tumor suppressor through promoting double strand break (DSB) repair by homologous recombination (Brzovic et al. 2003; Hashizume et al. 2001; Tarsounas and Sung 2020) . Several groups have shown that the early embryonic lethality of *brca1* mutant mice can be partially suppressed by mutation of the tumor suppressor *53bp1* , which promotes the error prone non-homologous end joining (NHEJ) pathway (Cao et al. 2009; Chen et al. 2020; Li et al. 2016) . In *C. elegans,* orthologs of BRCA1 and BARD1, ( *brc-1* and *brd-1 ,* respectively), also play roles in DSB repair but have only mild embryonic lethal phenotypes (Boulton et al. 2004; Janisiw et al. 2018; Li et al. 2018) . Additionally, analysis of the *53bp1* ortholog, *hsr-9* , did not reveal an obvious role in NHEJ (Ryu et al. 2013) . These results suggest that the function of *hsr-9* and relationship between *brc-1 - brd-1* and *hsr-9* may be different in this metazoan than in mammals.

We constructed *hsr-9 ; brc-1* and *hsr-9 ; brd-1* double mutants and analyzed embryonic lethality to examine the genetic interaction between these genes in *C. elegans* . In contrast to what has been reported in mice, we observed elevated embryonic lethality in *brc-1* and *brd-1* null alleles [ *brc-1 ( xoe4 ), brd-1 (xoe18)* (Li et al. 2023; Li et al. 2018) ], in combination with either *hsr-9 ( ok759 )* (Ryu et al. 2013) or a new putative null allele *hsr-9 (xoe17)* ( Figure 1A ). On the other hand, a hypomorphic *brc-1* allele, *brc-1 ( tm1145 )* (Li et al. 2018) , in combination with *hsr-9 (xoe17)* did not result in elevated embryonic lethality ( Figure 1A ). To determine whether the elevated embryonic lethality was due to *polq-1* -dependent microhomology-mediated end joining (MMEJ), which is mutagenic and activated in the absence of *brc-1* (Kamp et al. 2020) , we also constructed a new putative null allele of *polq-1* [ *polq-1 (xoe51)* ]. We found that the *hsr-9 ; brc-1 polq-1* triple mutant had levels of embryonic lethality similar to *brc-1 polq-1* but higher than *hsr-9 ; brc-1* , suggesting that the elevated embryonic lethality of *hsr-9 ; brc-1* is not a consequence of activation of MMEJ ( Figure 1B ). Therefore, our results are consistent with a model where BRC-1 - BRD-1 and HSR-9 function in parallel pathways to promote viable progeny, most likely through DSB repair, and do not appear to be antagonist as in mammals.

## Methods

**CRISPR-mediated genome editing:** *hsr-9 (xoe17)* and *polq-1 (xoe51)* alleles were engineered by incorporating the stop-in cassette (Wang et al. 2018) early in the coding region of each gene and were generated using the co-CRISPR method (Paix et al. 2015) . The *hsr-9* repair template (gattttgcctcttaaataaaatttcagCAAAAAACCGAGGGGAGACTTGCAATAGGGAAGTTTGTCCAGAGCAGAGGTGACTAAGTGATAAGCTAGCTCTCGGATCATCTTGCAAACATGCTTATTGCTGgtaggtattgcaacc) and guide RNA (AGGGGAGACTTGCAATATCT) were injection into N2 and the resulting progeny were analyzed by PCR using TGAAATTAAGGTGGTCACTCGAAG and GTTGTTGTGGGGAGGCTGAA. The *polq-1* repair template (AGAGAATTCTCTGAAGATCCATTAATATTGCTTACCGAAGGGGAAGTTTGTCCAGAGCAGAGGTGACTAAGTGATAAGCTAGCAGAGTTTTCGCCGCAATTCTCAGACTTTGGTAATGATTTC) and guide RNA (ATTGCGGCGAAAACTCTCTT) were injected into N2 and the resulting progeny were analyzed by PCR using ATAGGCAAATGGCTGGACGG and TCAAAGCAGTCTTCTCGGCA. Worms were outcrossed a minimum of three times.

**Embryonic lethality:** L4 hermaphrodites of indicated genotypes were picked onto individual plates and transferred to new plates every 24hr for 3 days. Embryonic lethality was determined by counting eggs and hatched larvae 24hr after removing the hermaphrodite and calculating percent as eggs/(eggs + larvae).

## Reagents

Strains:

**Table:** 

Strain	Genotype	Available from
N2	*Caenorhabditis elegans*	CGC
JEL730	*brc-1 ( xoe4 ) *	JE lab, deposited in CGC
JEL1000	*hsr-9 (xoe17) *	JE lab, will be deposited in CGC
WB240	*hsr-9 ( ok759 ) *	CGC
JEL1162	*brd-1 (xoe18) *	JE lab, will be deposited in CGC
JEL1016	*hsr-9 (xoe17); brc-1 ( xoe4 ) *	JE lab, will be deposited in CGC
JEL838	*hsr-9 ( ok759 ); brc-1 ( xoe4 ) *	JE lab
JEL1166	*hsr-9 (xoe17); brc-1 ( tm1145 ) *	JE lab
JEL1319	*hsr-9 (xoe17); brd-1 (xoe18) *	JE lab, will be deposited in CGC
JEL1134	*polq-1 (xoe51) *	JE lab, will be deposited in CGC
JEL1142	*hsr-9 (xoe17); brc-1 ( xoe4 ) polq-1 (xoe51) *	JE lab, will be deposited in CGC
JEL1104	*brc-1 ( xoe4 ) polq-1 (xoe51) *	JE lab
JEL1199	*hsr-9 (xoe17); polq-1 (xoe51) *	JE lab
